# Anti-Inflammatory and Immune Regulatory Actions of *Naja naja atra* Venom

**DOI:** 10.3390/toxins10030100

**Published:** 2018-02-28

**Authors:** Shu-Zhi Wang, Zheng-Hong Qin

**Affiliations:** 1 Institute of Pharmacy and Pharmacology, University of South China, Hengyang 421001, China; wangshuzhi727@126.com; 2 Hunan Province Cooperative Innovation Center for Molecular Target New Drug Study, University of South China, Hengyang 421001, China; 3 Department of Pharmacology and Laboratory of Aging and Nervous Diseases, Jiangsu Key Laboratory of Translational Research and Therapy for Neuro-Psycho-Diseases, Jiangsu Key Laboratory of Preventive and Translational Medicine for Geriatric Diseases, College of Pharmaceutical Science, Soochow University, Suzhou 215123, China

**Keywords:** cobra venom, anti-inflammation, immune regulation, T lymphocytes, complement system, NF-κB, rheumatoid arthritis, nephritis, pneumonia, systemic lupus erythematosus, allograft rejection

## Abstract

*Naja naja atra* venom (NNAV) is composed of various proteins, peptides, and enzymes with different biological and pharmacological functions. A number of previous studies have reported that NNAV exerts potent analgesic effects on various animal models of pain. The clinical studies using whole venom or active components have confirmed that NNAV is an effective and safe medicine for treatment of chronic pain. Furthermore, recent studies have demonstrated that NNAV has anti-inflammatory and immune regulatory actions in vitro and in vivo. In this review article, we summarize recent studies of NNAV and its components on inflammation and immunity. The main new findings in NNAV research show that it may enhance innate and humoral immune responses while suppressing T lymphocytes-mediated cellular immunity, thus suggesting that NNAV and its active components may have therapeutic values in the treatment of inflammatory and autoimmune diseases.

## 1. Introduction

Metabolic inflammation and autoimmune diseases, such as rheumatoid arthritis (RA), nephritis, chronic obstructive pulmonary disease, systemic lupus erythematosus (SLE), and multiple dermatosis, have become growing public health issues in recent years because of the high incidence and high rate of disability or death [1,2,3,4,5]. These diseases are mainly caused by excessive inflammatory and abnormal immune responses accompanied by inflammatory and immune cytokines secretion, which may lead to lesions and functional damage of normal tissues and cells. 

Cobra venom is a complex mixture of various components secreted by the poisonous glands of the cobra, such as *Naja naja atra*, *Naja kaouthia*, *Naja haje*, and others. More than 90% of substances comprising the lyophilized powder of cobra venom are proteins, which are also the main constitutes of biological activities and lethal toxicity. For a long time, a great deal of studies showed that cobra venom manifested a great role in relieving pain, for example, cancer pain, neuralgia, arthralgia, and so on [6,7,8]. ‘Cobratoxin injection’, which is extracted from cobra venom neurotoxin and is an approved drug, has been used for pain treatment in clinic for many years in China. Meanwhile, Chen et al. reported that the neurotoxin of cobra venom presented analgesic effects through the cholinergic system but not the opioid system [9]. Interestingly, in recent years, there were several studies showing that *Naja naja atra* venom (NNAV) had potent anti-inflammation and immune regulation effects [10,11]. NNAV is also effective in relieving symptoms and tissue damage in animal models of RA, SLE, and nephrotic syndrome [11,12,13]. These recent findings may suggest some new clinical applications of NNAV and its active components.

## 2. Composition and Characteristics of Cobra Venom

Cobra venom is a viscous liquid with light yellow color and consists of a mixture of proteins, peptides, and enzymes that have a variety of biochemical and pharmacological functions. The main components include neurotoxins, cardiotoxins (which are also called cytotoxins), phospholipase A_2_ (PLA_2_), cobra venom factor (CVF), nerve growth factor (NGF), and so on [14,15]. 

### 2.1. Neurotoxin 

Neurotoxins from cobra venom are a series of low molecular weight peptides that belong to the three-finger protein family, including several alpha-neurotoxins, which may possess high selectivity for nicotinic acetylcholine receptors (nAChRs) [16]. nAChRs are receptor proteins that react to neurotransmitter acetylcholine and mediate neurotransmissions in the central and peripheral nervous systems as well as neuro-muscle junctions [17]. Previous studies showed that nAChR α7 subunit was involved in the analgesia action of neurotoxin isolated from *Naja naja kaouthia* [18,19]. Owing to the low potential of drug tolerance and addiction, cobra venom neurotoxins have a broad prospect for pain management. 

α-Neurotoxins from cobra venom are usually the post-synaptic neurotoxins. The short-chain α-neurotoxins are composed of 60–62 amino acid residues and four disulfide bridges, while long-chain α-neurotoxins are composed of 66–75 amino acid residues and five disulfide bridges. Both short- and long-chain neurotoxins are selective for nAChRs [20,21]. Short-chain α-neurotoxins were reported to have three isoforms. Tu reported that a short-chain α-neurotoxin (atratoxin) purified from NNAV was identified to be an alkaline protein that possessed a molecular weight of 6952 Da with an isoelectric point of approximately 9.5 [22]. The main component of ‘Cobratoxin Injection’ (which is a Chinese approved drug) is an α-neurotoxin named cobratoxin, which is purified from cobra venom produced in Hunan province in China and is estimated to be a 7-kDa peptide. In our studies, a short-chain α-neurotoxin is isolated from NNAV produced in Guangxi and Jiangxi province. Its molecular weight is 6944 Da, as detected by mass spectrometry, and consists of 62 amino acid residues with no signal peptide. Its amino acid sequence is LECHNQQSSQTPTTTGCSGGETNCYKKRWRDHRGYRTERGCGCPSVKNGIEINCCTTDRCNN as determined by amino acid sequencing of whole peptide. This neurotoxin is consistent with that reported in gene database as cobrotoxin [23]. 

### 2.2. Cardiotoxin

Cardiotoxins from cobra venom are also called cytotoxins, which are approximately 40% to 50% of the dry weight in total cobra venom. Cardiotoxins were reported to be divided into six isoforms named CTX I, CTX II, CTX III, CTX IV, CTX V, and CTXn [24]. Previous research reported that cardiotoxins are basic proteins with isoelectric points larger than ten and with molecular weights that are approximately 6.5 kDa [25]. Dubovskii showed that cardiotoxins are also membrane-active proteins in the three-finger toxin family, which consist of about 60 amino acid residues and four disulfide bridges [26]. 

As the name implies, cardiotoxins are components that exert toxicity to heart, cancer cells, and even normal cells [27,28]. Furthermore, cardiotoxins possess various biological activities including depolarization of membranes and impairment of skeletal muscles, in addition to lysis of erythrocytes, lymphocytes, and epithelia cells [29,30,31,32]. However, Jiang et al. demonstrated that najanalgesin from *Naja naja atra*, which is homologous with cardiotoxin, possessed potent antinociceptive activities [33]. Meanwhile, cardiotoxin III purified from NNAV exhibited anticancer activities [34]. 

### 2.3. Phospholipase A_2_ (PLA_2_) 

PLA_2_s include both basic and acidic isoforms Basic PLA_2_ isoforms possess various toxicological actions such as cytotoxicity and hemolytic activity, while acidic isoforms exhibit higher enzymatic activities with no apparent toxic effects [35]. It was reported that PLA_2_ from *Ophiophagus hannah* was an acidic enzyme with an isoelectric point of 4.0 [36], and the molecular weight was measured to be 14 kDa by SDS-polyacrylamide gel electrophoresis [37], while the molecular weight of acidic PLA_2_ from *Naja naja* venom was estimated to be 15.2 kDa [38]. It is commonly known that PLA_2_ from different species exhibited anti-coagulant activities [38,39], suggesting its prospect for treatment and prevention of cardiovascular disorders. Nowadays, its neuroprotection, anti-apoptosis, and antibacterial [40,41] effects have also been observed. 

### 2.4. Cobra Venom Factor (CVF)

CVF is a three-chain protein that has a molecular weight of about 149 kDa, and consists of α-, β-, and γ-chains that are connected with disulfide bridges [42]. CVF purified from *Naja kaouthia* venom is a kind of acid glycoprotein and the isoelectric point is approximately 6.2; SDS-polyacrylamide gel electrophoresis shows the molecular weights of each of the chains are 65.4 kDa, 52.1 kDa, and 35.5 kDa, respectively [43]. A great deal of studies discovered that CVF was structurally and functionally similar to complement C3, and accordingly had the abilities of influencing the activation of complement system and development of immunological diseases [42,44]. Basically, CVF is able to deplete complement C3 and thus inhibit immune reaction and inflammation. 

### 2.5. Nerve Growth Factor (NGF)

NGF is one of the most important biological active agents in the nervous system, and it is also the essential nutrient for neuronal survival, nerve fibers growth, differentiation, and regeneration [45]. Franca et al. reported that NGF comprised only 0.1% to 0.5% of the crude cobra venoms [46]. The analysis of 22 amino acid residues in the N-terminal sequence of NGF extracted from NNAV revealed that it was similar to the NGFs from other venoms. The isoelectric point of NGF was discovered to be 7.2 and the molecular weight was approximately 13.5 kDa [47]. 

Chen et al. reported that NGF isolated from NNAV displayed therapeutic actions on damaged sciatic nerves in adult cats [48]. Besides the neuronal protection, NGF was also demonstrated to increase plasma extravasation and histamine release from whole blood cells, which may explain the immunomodulatory effects in inflammatory events [49]. Furthermore, Angeletti declared that NGF from *Naja naja* presented no toxic effect both in vitro and in vivo [50]. 

## 3. Effect of NNAV and Its Components on Inflammation and Immune Activity

### 3.1. Anti-Inflammatory Actions

Animal inflammatory models are routinely used for the evaluation of drug effects. One commonly used, valid, and reliable rodent model is the formalin-induced inflammatory pain experiment [51]. Shi et al. reported that cobratoxin, a long-chain α-neurotoxin from *Naja naja kaouthia* venom, decreased formalin-induced licking responses in rats in both the early (0–15 min after formalin injection) and late (20–60 min after formalin injection) phases [19]. Meanwhile, NNAV presented similar results in mice, suggesting that cobra venom could ameliorate inflammation [11]. In addition, in egg-white-induced non-specific inflammation and formalin-soaked filter papers triggered granuloma rat models, which are also frequently used for anti-inflammatory assays [52,53], NNAV relieved rat foot edema and decreased granuloma proliferation [11]. In another study, Zhu et al. demonstrated that cobrotoxin, a short-chain neurotoxin form NNAV, ameliorated body inflammatory reactions on three rodent experimental inflammation models: formaldehyde-induced mouse foot swelling test, mouse abdominal capillary osmosis test, and Whatman paper-inducing rat granuloma formation test [54]. Cardiotoxin from NNAV also presented anti-inflammatory actions on formalin test, egg-white-induced non-specific inflammation test, formalin-soaked filter papers triggered granuloma test, and abdominal capillary osmosis test [55]. These studies illustrated that cobra venom and neurotoxins possessed anti-inflammatory capabilities. 

Nuclear factor-κB (NF-κB) is a transcriptional factor that regulates inflammatory responses in various conditions [56,57,58]. Under normal states, NF-κB binds to inhibitory IκB proteins (IκB-𝛼, IκB-β, IκB-ε, etc.) in the cytoplasm [59]. Under inflammatory conditions, pro-inflammatory cytokines such as tumor necrosis factor (TNF-α) and interleukin-1β (IL-1β) may activate IκB kinases (IKKs) phosphorylation [60]. When the P-IKKs are upregulated, the IκB proteins are degraded by the proteasomes, which will then trigger the translocation of liberated NF-κB to the nucleus to activate target gene transcription including TNF-α, interleukins, and so on [61]. 

Previous studies demonstrated that NNAV decreased P-IKK-α levels and upregulated IκB-α expression, thus blocking the nuclear translocation of NF-κB p65. Meanwhile, NNAV reduced TNF-α and IL-1β levels in an adriamycin-induced rat nephropathy model [12]. In another study, Wang et al. proved that cobrotoxin (the neurotoxin from NNAV) reduced P-IκB-α expression and elevated the level of IκB-α, while also inhibiting NF-κB p65 nuclear translocation in an adriamycin-induced rat nephropathy model [62]. In addition, Ruan et al. [63] found that Neurotoxin-Nna, which is separated from NNAV, inhibited NF-κB activation and decreased TNF-α, IL-1β, inducible nitric oxide synthase (iNOS), intercellular adhesion molecule-1 (ICAM-1), myeloperoxidase (MPO), and malondialdehyde (MDA) levels. Furthermore, cobrotoxin from *Vipera lebetina turanica* was reported to conjugate with IKKs and NF-κB p50 with high affinity, resulting in NF-κB signaling pathway inhibition. Moreover, the cobrotoxin downregulated lipopolysaccharide (LPS)-evoked TNF-α, interleukin-4 (IL-4), cydooxygenase-2 (COX-2), and iNOS expression in astrocytes [64]. The results from all of these studies suggest that cobra venom and its active components might suppress inflammatory responses through inhibiting the NF-κB signaling pathway. 

### 3.2. Immune Regulatory Actions

The immune system can protect the body against foreign microorganisms and abnormal cells infringement [65]. On the one hand, immune deficiency such as T lymphocytes absence or HIV invasion leads to repeated and abnormal infections [66]. On the other hand, immune overreaction causes damages to body tissues and cells. For instance, autoimmune diseases such as RA and SLE are generated when the immune systems reacts to autoantigens [67]. 

Kou et al. reported that NNAV had immune regulatory effects in different conditions. In mice with normal immunity, NNAV increased natural killer cell activity, B lymphocytes proliferation, and sheep red blood cell (SRBC)-induced antibody production in various immune animal models. Otherwise, NNAV suppressed mice delayed-type hypersensitivity reaction evoked by dinitrofluorobenzene, and inhibited T lymphocyte proliferation that was stimulated by concanavalin A. inhibited T lymphocyte proliferation, which was stimulated by concanavalin A. In dexamethasone-caused low-immunity mice, NNAV restored serum IgG and IgM levels and helped the recovery of the spleen germinal center. Meanwhile, in mice spleen cell experiments, NNAV inhibited CD4^+^ T and CD8^+^ T cells proliferation examined with flow cytometry. Unexpectedly, NNAV selectively elevated Th1 and Th2 cytokines such as interferon-γ (IFN-γ) and IL-4 production and reduced Th17 cytokine interleukin-17 (IL-17) secretion. Hence, all these results manifested that NNAV reinforced innate and humoral immune responses while inhibiting T lymphocyte-mediates cellular immunity, and the latter was usually correlated with overreacted inflammatory and immune responses in auto-immune diseases [10]. 

Another study performed by Xu et al. [68] evaluated the effects of NNAV and its component neurotoxin on skin allograft rejection in rats. As we know, acute rejection occurs several days or weeks after skin transplantation, which is induced by cytotoxic T cells, monocytes, or macrophages [69,70]. Nowadays, corticosterone, cyclosporine A, or cytotoxic drugs are used clinically for acute rejection treatment, while they also produce severe side effects that may increase the probability of tissue damage, infections, or malignant tumors [71]. For this reason, a new safe and effective agent for skin transplant rejection is sorely needed. Interestingly, Xu [68] found that NNAV and cobrotoxin extended skin allograft retention time in rats. In histological sections of skin, spleen, and thymus, NNAV and cobrotoxin inhibited inflammatory cells infiltration and recuperated the germinal center in immune organs. Meanwhile, NNAV and cobrotoxin decreased rejection-induced T lymphocytes proliferation and reduced the CD4^+^ T/CD8^+^ T proportion. Furthermore, cobrotoxin inhibited T lymphocytes proliferation via influencing the cell cycle at G0/G1 phase. This study suggested that NNAV and cobrotoxin restrained skin allograft rejection by suppressing T cell-mediated immune responses. 

The complement system plays important roles in the innate and adaptive immune system that involves the recognition and resistance of various invading pathogens [72,73]. Complement C3 was reported to be the key modulator in the complement system that promoted transplant rejection, which led to impairment of organs and tissues [74]. A previous study discovered that CVF was structurally and functionally similar to complement C3, and it may suppress the complement system activation [75]. Indeed, Oberholzer et al. found that CVF reduced C3 deposition and prolonged the survival time of the xenograft pancreatic islets in a rat to mouse model [76]. Another study demonstrated that CVF suppressed hyper-acute xenograft rejection and prolonged survival of guinea-pig-to-rat heart xenografts via depleting complement [77]. Additionally, CVF was reported to deplete complement and delay acute cell-mediated rejection in pig-to-mouse corneal xenotransplantation. Meanwhile, CVF also inhibited macrophages and CD4^+^ T cells infiltration and prolonged the porcine corneal xenograft survival [78]. Li et al. demonstrated that CVF pre-treatment reduced acute complement-dependent antibody-mediated humoral rejection after allograft cardiac transplantation in rats [79]. These studies suggested that CVF may ameliorate transplant rejection through depleting complement and suppressing humoral and cellular immune reactions. 

## 4. Research of NNAV and Its Components on Inflammatory and Immune Diseases

### 4.1. Effects on Rheumatoid Arthritis

RA is an autoimmune disease that is characterized by symmetric and chronic inflammatory pathology of several joints [80]. Patients with RA show symptoms of joint pain, swelling, and functional impairment, which may at last lead to joint stiffness, malformation, and disability [81]. The pathogenesis of RA is related to genetic susceptibility, physical stress, abnormal hormone levels, immune status, and environment [1]. Nonsteroidal and steroidal anti-inflammatory drugs are usually used for RA treatment in clinic [82], while the prognosis and side effects of these medicines are unsatisfactory. Hence, drugs with potent efficacy and lower adverse effects are in extreme demand. 

Gomes demonstrated that *Naja kaouthia* venom presented anti-arthritic activity in Freund’s complete adjuvant (FCA)-induced arthritis in rats [83]. This study found that *Naja kaouthia* venom decreased paw and ankle volume and ameliorated urinary hydroxyproline, glucosamine, serum acid, and alkaline phosphatase levels. Liu et al. reported that cobratoxin, the long-chain α-neurotoxin from *Naja naja kaouthia* venom, also exerted therapeutic effects on adjuvant arthritis in rats [84]. In the research of NNAV, Zhu [11] proved that NNAV ameliorated paw edema and pathological lesions in adjuvant arthritis rats, and NNAV also reduced serum TNF-α levels and elevated serum interleukin-10 (IL-10) concentrations, indicating that NNAV may relieve adjuvant arthritis via attenuating inflammation. Furthermore, Chen [55] demonstrated that cardiotoxin from NNAV administrated to rats obtained a significantly lower mean arthritic score versus model adjuvant arthritis rats in pathological sections. Meanwhile, cardiotoxin decreased serum pro-inflammatory cytokines interleukin-6 (IL-6) and IL-17 expressions, while selectively inhibiting CD4^+^ T subsets on peripheral blood cells in adjuvant arthritis rats. In another study performed by Zhu [54], cobrotoxin, the neurotoxin abstracted from NNAV, also inhibited pro-inflammatory cytokines secretion and T cells proliferation. In addition, the research demonstrated that cobrotoxin suppressed NF-κB signaling pathway activation in fibroblast-like synoviocytes. Accordingly, these consequences suggested that cobra venom from different species produced anti-inflammatory and immune- regulation effects on RA. 

### 4.2. Effects on Acute and Chronic Nephropathy 

Acute and chronic nephropathy are becoming serious global health problems due to high prevalence and fatality [85,86]. Previous research reported that NNAV presented protective effects on glycerol-induced acute renal failure and adriamycin-triggered nephrotic syndrome in rats [12]. As we know, acute nephropathy leads to the rapid alteration of renal function, while chronic nephrotic syndrome shows symptoms of hypoalbuminemia, hyperlipidemia, persistent proteinuria, edema, and hypertension [87,88]. Nowadays, serum creatinine (SCr), blood urea nitrogen (BUN), and serum cystatin C (Cys-C) are measured to evaluate renal function in clinic [89,90]. Wang et al. demonstrated that NNAV ameliorated twenty-four-hour urine protein excretion, decreased SCr, BUN, and Cys-C levels, reduced serum total cholesterol and triglyceride content, and recovered serum albumin levels. Meanwhile, NNAV markedly ameliorated renal pathological lesions that were observed with light microscopy and transmission electron microscopy. Furthermore, NNAV may ameliorate acute and chronic nephropathy through improving body oxidative stress and inhibiting the NF-κB signaling pathway [12]. Afterwards, neurotoxin and cardiotoxin from NNAV were also reported to manifest protective effects on adriamycin-evoked nephrotic syndrome in rats [62,91]. 

Diabetic nephropathy is considered to be the capital cause of end-stage renal disease [92]. Dai et al. showed that NNAV reduced blood glucose level and SCr, BUN, serum Cys-C, total cholesterol, and triglyceride levels in diabetic nephropathy rats, meanwhile, NNAV also decreased urinary protein excretion and *N*-acetyl-β-glucosaminidase concentration together with ameliorated glomerular damages in pathological sections [93]. This study revealed that NNAV may also exert protective effects on diabetic nephropathy in rats.

### 4.3. Effects on Acute Lung Injury and Pulmonary Fibrosis

Nowadays, the incidence of pulmonary diseases continues to grow, owing largely to atmospheric pollution and environment alterations [94,95]. In respiratory diseases, acute inflammatory reactions and recruitment of immune cells such as neutrophils, monocytes, and macrophages were reported to occur in the lesion areas [96], followed by production and secretion of large amounts of reactive oxygen species and pro-inflammatory cytokines such as TNF-α, IL-1β, and IL-6, which may provoke alveolar damage and obstruct gas exchange [97,98]. Cui et al. [99] illustrated that NNAV ameliorated lung gas-exchange dysfunction, and restored the histopathological changes in rat lung tissue sections stained with HE and Masson staining. In addition, NNAV elevated superoxide dismutase (SOD) and glutathione (GSH) levels while downregulating MDA levels. Meanwhile, NNAV decreased serum TNF-α and IL-1β levels and inhibited NF-κB signaling pathway activation. In addition, inhibition of transforming growth factor-β (TGF-β)/Smad pathway was also reported to be associated with the properties of NNAV. This study by Cui proved that NNAV may improve lung injuries via inhibiting oxidative stress and inflammatory reactions. Interestingly, in our further research, neurotoxin from NNAV also exerted protective effects on LPS-induced pulmonary injuries by inhibiting bronchoalveolar lavage fluid (BALF) immune cells including macrophages and lymphocytes accumulation and pro-inflammatory cytokines secretion, meanwhile, the neurotoxin also reduced oxidative stress and the CD4^+^ T/CD8^+^ T ratio (unpublished data). 

CVF was reported to have potent effects on suppressing pulmonary collagen deposition via complement depletion in bleomycin-triggered lung fibrosis rats [100]. Ren demonstrated that CVF separated from NNAV ameliorated pulmonary injuries evoked by oleic acid, and CVF also improved pulmonary function by reversing hypoxemia, dynamic compliance reduction, and pulmonary blood flow decrease [101]. Another study performed by Mao et al. proved that CVF pretreatment ameliorated acute pulmonary injury induced by intestinal ischemia-reperfusion in rats. In this study, CVF decreased oxidative stress, neutrophils infiltration, ICAM expression, and interleukin-8 (IL-8) secretion [102]. All these findings revealed that cobra venom and its components may be novel therapeutic agents for the treatment of chronic inflammatory pulmonary diseases. 

### 4.4. Effects on Systemic Lupus Erythematosus

SLE is an autoimmune disease that mostly affects females aged twenty to forty, which is typified by superabundant autoantibodies generation and immune complex deposition in multiple organs such as skin, small blood vessels, kidney glomerulus, and joints [103]. The pathogenesis of SLE is uncertain; a great number of studies indicated that SLE development was related to heredity, endocrine abnormality, infection, immune disorder, and environment factors [104,105,106]. Zhu et al. [13] reported that NNAV ameliorated skin erythema, proteinuria excretion, and pathological lesions in spleen and kidneys in MRL/lpr SLE mice. Emotional disturbances and nervous system changes were also reported in SLE patients [107]. NNAV reduced mice anxiety-like behaviors observed in an inner open field test. Furthermore, NNAV decreased anti-dsDNA antibody levels and the concentrations of pro-inflammatory cytokines TNF-α and IL-6, suggesting that NNAV inhibited auto-immune and inflammatory responses in SLE mice [13]. 

## 5. Safety Considerations of NNAV and Cobrotoxin

In our mice experiments with Bliss assays, we determined that the lethal dose 50 (LD50) of NNAV by oral administration, intraperitoneal injection, and intravenous injection were 102.3 mg/kg, 996.6 μg/kg, and 623.9 μg/kg, respectively. The 95% confidence limit ranges were 84.9–123.3 mg/kg, 900.6–1102.8 μg/kg, and 572.3–680.2 μg/kg, respectively. In another experiment, we measured that the LD50 of cobrotoxin (the neurotoxin from NNAV) by subcutaneous injections was 59.991 μg/kg, and the 95% confidence limit range was 57.473–61.846 μg/kg. The LD50 of cobrotoxin in oral administration was approximately 90 μg/kg. In the previous studies, we found that NNAV may exert anti-inflammatory and immune regulatory effects at the dosages of 20–300 μg/kg by oral administration. Meanwhile, cobrotoxin may produce anti-inflammatory and immune regulatory effects in the dosages of 5–45 μg/kg by oral administration and 2.5–10 μg/kg by subcutaneous injections. These preliminary safety data suggest that clinical use of NNAV and cobrotoxin are safe.

## 6. Conclusions and Perspectives

NNAV and its active components presented anti-inflammatory and immune regulatory effects on various rodent models via strengthening innate and humoral immune responses, inhibiting T lymphocyte-mediated cellular immunity, influencing complement system, and suppressing NF-κB signaling pathway activation. These outcomes suggest that NNAV and its active components might represent new medicines for inflammatory and auto-immune diseases, which are difficult to cure at present. Cobra venoms have been used for relieving various kinds of pain in clinic in China for many years. In recent years, researchers have found that cobra venoms are effective for the treatment of chronic pain, neuralgia, rheumatoid arthritis, herpes infection, neurodermatitis, and chronic kidney disease; additionally, doctors practicing oriental medicine in Korea have used cobra venoms to treat diabetes patients. Meanwhile, adverse reactions were rarely found under therapeutic dosages (usually 1–5 μg by acupoint injection, unpublished observations). These results suggest that cobra venom and its active components may have unique value for the treatment of some incurable diseases. 

## Abbreviations

NNAV	*Naja naja atra* venom
RA	rheumatoid arthritis
SLE	systemic lupus erythematosus
PLA2	phospholipase A2
CVF	cobra venom factor
NGF	nerve growth factor
nAChRs	nicotinic acetylcholine receptors
NF-κB	nuclear factor-κB
TNF-α	tumor necrosis factor
IL-1β	interleukin-1β
IKKs	I𝜅B kinases
iNOS	inducible nitric oxide synthase
ICAM-1	intercellular adhesion molecule-1
MPO	myeloperoxidase
MDA	malondialdehyde
LPS	lipopolysaccharide
IL-4	interleukin-4
COX-2	cydooxygenase-2
SRBC	sheep red blood cell
IFN-γ	interferon-γ
IL-17	interleukin-17
FCA	Freund’s complete adjuvant
IL-10	Interleukin-10
IL-6	Interleukin-6
SCr	serum creatinine
BUN	blood urea nitrogen
Cys-C	serum cystatin C
SOD	superoxide dismutase
GSH	glutathione
TGF-β	transforming growth factor-β
BALF	bronchoalveolar lavage fluid
IL-8	interleukin-8
LD50	lethal dose 50
